# Structural basis of ethnic-specific variants of PAX4 associated with type 2 diabetes

**DOI:** 10.1038/s41439-021-00156-8

**Published:** 2021-07-05

**Authors:** Jun Hosoe, Ken Suzuki, Fuyuki Miya, Takashi Kato, Tatsuhiko Tsunoda, Yukinori Okada, Momoko Horikoshi, Nobuhiro Shojima, Toshimasa Yamauchi, Takashi Kadowaki

**Affiliations:** 1 Department of Diabetes and Metabolic Diseases, Graduate School of Medicine, The University of Tokyo, Tokyo, Japan; 2 Department of Statistical Genetics, Osaka University Graduate School of Medicine, Osaka, Japan; 3 Department of Medical Science Mathematics, Medical Research Institute, Tokyo Medical and Dental University, Tokyo, Japan; 4 Laboratory for Medical Science Mathematics, RIKEN Center for Integrative Medical Sciences, Yokohama, Japan; 5 CREST, JST, Tokyo, Japan; 6 Laboratory for Medical Science Mathematics, Department of Biological Sciences, Graduate School of Science, The University of Tokyo, Tokyo, Japan; 7 Laboratory for Genomics of Diabetes and Metabolism, RIKEN Center for Integrative Medical Sciences, Yokohama, Japan; 8 Department of Prevention of Diabetes and Lifestyle-Related Diseases, Graduate School of Medicine, The University of Tokyo, Tokyo, Japan; 9 Department of Metabolism and Nutrition, Teikyo University Mizonokuchi Hospital, Kawasaki, Kanagawa Japan; 10 Toranomon Hospital, Tokyo, Japan

**Keywords:** Genome-wide association studies, Sequence annotation

## Abstract

Recently, we conducted genome-wide association studies of type 2 diabetes (T2D) in a Japanese population, which identified 20 novel T2D loci that were not associated with T2D in Europeans. Moreover, nine novel missense risk variants, such as those of *PAX4*, were not rare in the Japanese population, but rare in Europeans. We report in silico structural analysis of ethnic-specific variants of *PAX4*, which suggests the pathogenic effect of these variants.

Large-scale meta-analyses of genome-wide association studies (GWAS) of type 2 diabetes (T2D) have been performed recently^1–4^. In particular, Mahajan et al.^3^ reported T2D GWAS meta-analysis data on ~900,000 individuals of European ancestry, increasing the association signals in over 240 loci associated with T2D. Recently, we conducted an extensive T2D GWAS meta-analysis in a Japanese population and identified 28 new loci^4^, highlighting the value of genetic research in ethnically diverse populations. As an example of clinical differences between ethnic populations, East Asians are more likely to develop T2D at a lower body mass index than Europeans^5^, who have been reported to show a higher insulin response and lower insulin sensitivity.

We demonstrated that the biological pathway of the maturity-onset diabetes of the young (MODY), monogenic diabetes characterized by reduced β-cell function, showed the most significant association with T2D in both Japanese and European populations^4^, by performing pathway analysis of GWAS summary data in the two populations^1,4^ using Pascal software. Here we focused on protein-coding genes involved in the MODY pathway mapping nearest to lead variants at T2D loci, as previously reported^6^, and investigated ethnic differences in the associations of these loci with T2D between Japanese and European populations^3,4^. As suggested by a previous study^4^, most of these lead variants at T2D loci in either the Japanese or European population showed at least nominally significant (*P* < 0.05) associations in the alternative population, even if not genome-wide significant, except for variants that were rare or monomorphic in one population such as *NKX6-1* rs201597274, *HNF1A* rs187150787, and *PAX4* rs2233580 in the Japanese population and *HNF1A* rs56348580 in the European population. Interestingly, there were several loci such as *GCK* where lead variants in these populations were independent of each other (Supplementary Tables 1 and 2). As an example of a locus specific to East Asians, a previously unreported missense T2D variant of *PAX4* NM_006193:c.574C>A:p.(Arg192Ser) reached genome-wide significance^4^. This variant was located at the same amino acid as another established independent T2D variant NM_006193:c.575G>A:p.(Arg192His) (Table 2 in ref. ^4^). These two *PAX4* variants were not in linkage disequilibrium (JPT: *r*^2^ < 0.01). *PAX4* encodes a transcription factor that is important for β-cell development, and rare mutations of this gene are suggested to cause a subtype of MODY (OMIM #612225). Arg192 of PAX4 is located in the homeodomain, which is a DNA-binding domain conserved in a large family of transcription factors. Barrera et al.^7^ reported that PAX4 variants Arg192His and Arg192Ser caused alteration in DNA-binding specificity in experiments using protein-binding microarrays. In addition, it was reported that the PAX4 p.Arg192His mutant demonstrated decreased repression of the transcription of target genes involved in the maintenance of β-cell function compared with wild-type PAX4 in an in vitro study^8^. We annotated PAX4 variants Arg192His and Arg192Ser with prediction tools via wANNOVAR (http://wannovar.wglab.org/), which suggested that these variants would be deleterious (Supplementary Table 3). Furthermore, we conducted an in silico structural analysis of the pathogenic effect of these *PAX4* variants, which revealed a decrease of the PAX4 homeodomain stability and reduced DNA binding by this domain in both variants (Figs. 1, 2 and Supplementary Note).

**Fig. 1:**
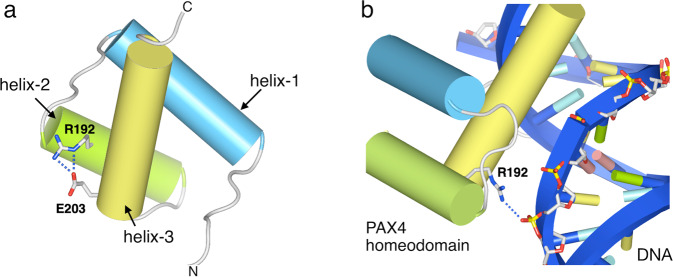
Structural model of the homeodomain in human PAX4. **a** The human PAX4 homeodomain is represented as cylinders, with the Arg192 and Glu203 residues being shown as sticks. The PAX4 homeodomain consists of three helices: helix-1 (light-blue), helix-2 (green), and helix-3 (yellow). Arg192 located on helix-2 is predicted to form salt-bridges with Glu203 located on helix-3. **b** Binding of the human PAX4 homeodomain to double-stranded DNA. DNA is shown by ribbon representation in blue. Arg192 of PAX4 (sticks) is predicted to bind with the phosphate backbone (sticks) of DNA.

**Fig. 2:**
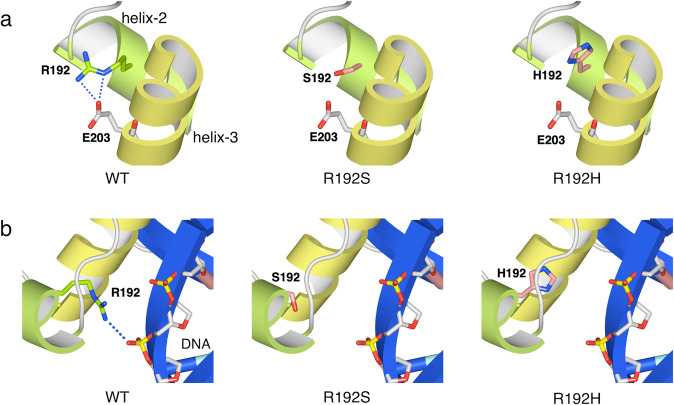
Structural analysis of two PAX4 variants, p.Arg192Ser, and p.Arg192His. PAX4 are shown by ribbon representation, with the key residues of PAX4 as sticks. The helix-2 and the helix-3 of PAX4 are shown in green and yellow, respectively. Wild-type Arg192 is green, while the Ser192 and His192 mutants are light red. **a** In wild-type PAX4, Arg192 forms salt-bridges with Glu203 (dotted blue line). In both mutants (p.Arg192Ser and p.Arg192His), the formation of salt-bridges with Glu203 is disrupted. **b** DNA is shown by ribbon representation in blue, with the phosphate backbone as sticks. In wild-type PAX4, Arg192 directly binds to the phosphate backbone of DNA (dotted blue line). In both mutants (p.Arg192Ser and p.Arg192His), binding with the phosphate backbone of DNA is disrupted.

We performed a functional study of INS-1 832/13 cells to assess the impact of these PAX4 variants, as described in Supplementary Note. Briefly, INS-1 832/13 cells were transfected with an equal amount of plasmid containing PAX4 wild-type and variants (p.Arg192Ser and p.Arg192His). Then, total RNA obtained from the INS-1 832/13 cells was reverse-transcribed into cDNA using the TaqMan Fast Advanced Cells-to-CT Kit (ThermoFisher SCIENTIFIC). Quantitative PCR was performed using the QuantStudio 7 Flex Real-Time PCR System (ThermoFisher Scientific). In the previous reports, PAX4 was suggested to maintain the integrity of the endoplasmic reticulum (ER) thus protecting β-cells from apoptosis^9^ as well as promoting the expression of the genes involved in β-cell survival, such as *Calr* encoding for calreticulin, a Ca^2+^ chaperone of the ER^8^. In the present study, we demonstrated that the transcription levels of *Calr* were decreased not only in INS-1 832/13 cells overexpressing PAX4 p.Arg192His, as previously reported^8^, but also in those overexpressing p.Arg192Ser, compared to those overexpressing wild-type PAX4 (Supplementary Fig. S1), which also suggested a pathogenic role for the *PAX4* variants.

To physiologically classify T2D loci, cluster analyses have been performed using data on metabolic traits^1,2,10^. Mahajan et al.^2^ performed a hierarchical cluster analysis of loci associated with T2D in ethnically diverse populations, generating three main clusters associated with BMI/dyslipidemia, insulin secretion, and insulin action. The cluster related to reduced insulin secretion contained a number of important loci involved in β-cell function as shown in their Supplementary Fig. 6^2^, with several lead variants, such as those of *PAX4* (p.Arg192His) and *HNF1A*, that showed distinct minor allele frequency (MAF) spectra and ethnically different associations (Supplementary Tables 10 and 11 in ref. ^2^). Such differences could lead to distinct proportions of the clusters of T2D loci with different underlying biological pathways^2,10^, which may result in variable proportions of subgroups of T2D patients classified based on their genetics in ethnically diverse populations. For example, the *PAX4* variants are specific to East Asians^4^, which presumably increase the proportion of the cluster of T2D loci with biological pathways influencing β-cell function in East Asian populations.

In a recent review article^11^, the pronounced ethnic heterogeneity associated with ethnic-specific risk variants (e.g., *PAX4* p.Arg192His and *HNF1A* p.Glu508Lys^12^) was suggested to be relatively unusual and not to explain observed ethnic differences in the presentation of T2D. It was also suggested that rare ethnic-specific variants identified through sequencing studies may be more important in this regard^11^. Although T2D GWAS meta-analysis recently performed in a Japanese population identified no less than 20 novel T2D loci^4^ that were not associated with T2D in Europeans^1,3^ (Supplementary Table 3 in ref. ^4^). Ethnic differences in the associations of a number of new loci with T2D may be affected by several factors, such as differences in allele frequency and diverse patterns of linkage disequilibrium in these populations as suggested recently^13^. For example, we identified nine novel missense variants such as those of *PAX4* (p.Arg192Ser) and *GLP1R* (p.Arg131Gln)^4^ that were in linkage disequilibrium with the lead variants of T2D loci and not rare in the Japanese population, but rare or monomorphic in Europeans (Supplementary Table 6 in ref. ^4^).

Considering the differences in sample size between these studies^3,4^, more large-scale genetic studies of East Asian populations, such as a recently reported extensive T2D GWAS meta-analysis^14^, are needed to fully elucidate similarities and differences in the pathogenesis of T2D between them. In conclusion, our new GWAS data^4^ suggested that large-scale single ethnic genetic studies could be useful for identifying ethnic-specific risk variants including relatively common ones, providing new insights into the genetic heterogeneity of T2D in diverse ethnic groups. The new information thus obtained would facilitate the development of tailored therapy for this disease in various populations.

## Supplementary information

Supplementary Figure 1

Clean version of Supplementary Data

## Data Availability

The relevant data from this Data Report are hosted at the Human Genome Variation Database at: 10.6084/m9.figshare.hgv.3018 and 10.6084/m9.figshare.hgv.3021.
