# Supplementary material for: Structural basis of ethnic-specific variants of PAX4 associated with type 2 diabetes
Source: Hum Genome Var. 2021 Jul 5;8:25. doi: 10.1038/s41439-021-00156-8 (PMC8257626; doi:10.1038/s41439-021-00156-8)
Supplement: Supplementary file 1 — Supplementary Figure 1 [file 41439_2021_156_MOESM1_ESM.pdf]

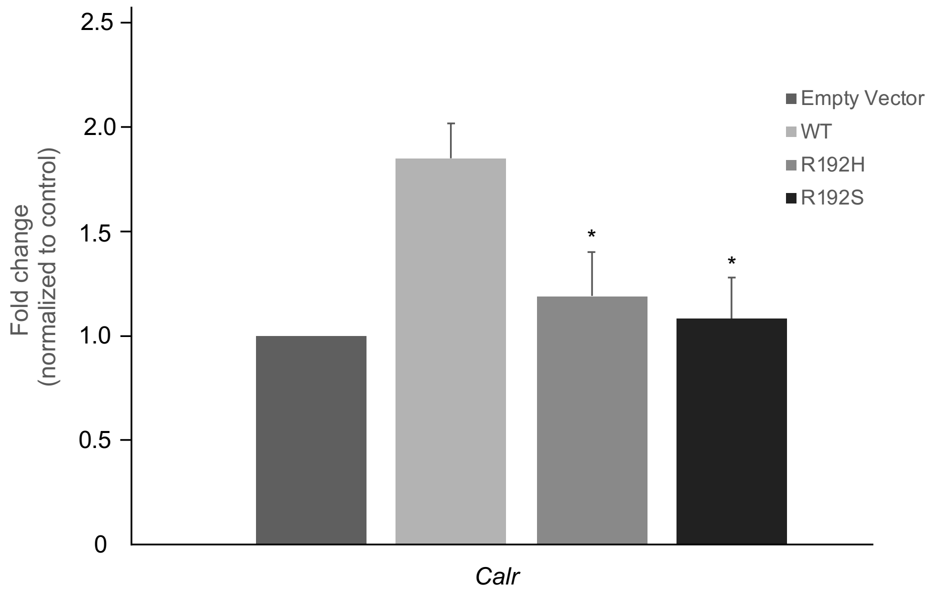

Supplementary Figure S1. Assessment of mRNA expression level of Calr in cells overexpressing PAX4 wild-type and variants. The *Calr* gene was assessed for its mRNA expression in INS-1 832/13 cells overexpressing PAX4 wild-type and variants using qPCR, with its relative mRNA expression calculated through normalization to the *GAPDH* mRNA level. The fold increase of the mRNA expression in the cells overexpressing PAX4 wild-type and variants compared with that in the cells transfected with empty vector was calculated and was shown as mean ± s.e.m. from four independent experiments. *$P < 0.05$ vs. wild-type.
