# Supplementary material for: Structural basis of ethnic-specific variants of PAX4 associated with type 2 diabetes
Source: Hum Genome Var. 2021 Jul 5;8:25. doi: 10.1038/s41439-021-00156-8 (PMC8257626; doi:10.1038/s41439-021-00156-8)
Supplement: Supplementary file 2 — Clean version of Supplementary Data [file 41439_2021_156_MOESM2_ESM.pdf]

Supplementary Information

Structural basis of ethnic-specific variants of PAX4 associated with type 2 diabetes

Jun Hosoe[1], Ken Suzuki[1,2], Fuyuki Miya[3,4,5], Takashi Kato[1], Tatsuhiko Tsunoda[3,4,5,6], Yukinori Okada[2], Momoko Horikoshi[7], Nobuhiro Shojima[*1], Toshimasa Yamauchi[*1], Takashi Kadowaki[*1,8,9,10]

1. Department of Diabetes and Metabolic Diseases, Graduate School of Medicine, The University of Tokyo, Tokyo, Japan
2. Department of Statistical Genetics, Osaka University Graduate School of Medicine, Osaka, Japan
3. Department of Medical Science Mathematics, Medical Research Institute, Tokyo Medical and Dental University, Tokyo, Japan
4. Laboratory for Medical Science Mathematics, RIKEN Center for Integrative Medical Sciences, Yokohama, Japan
5. CREST, JST, Tokyo, Japan
6. Laboratory for Medical Science Mathematics, Department of Biological Sciences, Graduate School of Science, The University of Tokyo, Tokyo, Japan
7. Laboratory for Genomics of Diabetes and Metabolism, RIKEN Center for Integrative Medical Sciences, Kanagawa, Japan
8. Department of Prevention of Diabetes and Lifestyle-Related Diseases, Graduate School of Medicine, The University of Tokyo, Tokyo, Japan
9. Department of Metabolism and Nutrition, Teikyo University Mizonokuchi Hospital, Kawasaki, Kanagawa, Japan
10. Toranomon Hospital, Tokyo, Japan

*Corresponding author:

Correspondence should be addressed to Nobuhiro Shojima, Toshimasa Yamauchi, and Takashi Kadowaki.

Supplementary Note

Building of structural models of two PAX4 mutants (p.Arg192Ser and p.Arg192His)

A 3-dimensional structural model of the human PAX4 homeodomain (amino acid residues 162-220) was constructed by homology modeling with SWISS-MODEL[15], using the X-ray crystal structure of human PAX3 (UniProt: P23760) (PDB ID: 3CMY)[16] as a template. Amino acid identity was 50.85% between the PAX4 and PAX3 homeodomains. When the quality of the structural model was evaluated by using QMEAN, the QMEAN Z-score of the PAX4 model was -0.55, indicating that the model was reliable[15]. A structural model of the PAX4 homeodomain binding with DNA was built from PAX3 complexed with DNA (PDB ID: 3CMY)[16] by superimposing PAX4 onto PAX3. Superimposition and merging of coordinate data were performed with Waals (Altif Laboratories, Inc., Tokyo, Japan). Structural models of two PAX4 mutants (p.Arg192Ser and p.Arg192His) were built by using the mutation and energy minimization command of Swiss-Pdb Viewer[17]. Comparison between the structural models of wild-type PAX4 and each mutant was performed with Waals. Coordinate data for the X-ray crystal structures of the human PAX3 (PDB ID: 3CMY)[16] was obtained from the Protein Data Bank: PDB (https://www.rcsb.org). A sequence of *PAX4* (UniProt: O43316) was obtained from the UniProt database (https://www.uniprot.org/).

Analysis of structural modifications in the PAX4 mutants (p.Arg192Ser and p.Arg192His)

T2D GWAS meta-analysis recently performed in a Japanese population showed that a previously unreported missense variant of *PAX4* (p.Arg192Ser) reached genome-wide significance[4]. Another previously established independent T2D variant of *PAX4* (p.Arg192His)[18] located at the same amino acid was reported to be associated with reduced C-peptide levels in a genetic study of T2D in Korea[19]. It was reported that an *in vitro* study of the PAX4 p.Arg192His mutant demonstrated impaired repression of the transcription of target genes involved in maintenance of β-cell function compared with wild-type PAX4[8]. To investigate the influence of these *PAX4* mutations, we constructed 3-dimensional structural models of the wild-type human PAX4 homeodomain and the two mutants (Arg192Ser and Arg192His). Then we compared the model of each mutant with that of wild-type PAX4 to identify structural defects. The homeodomain of PAX4 is a conserved DNA-binding module that consists of three α-helices (helix-1, helix-2 and helix-3), and helix-3 of the homeodomain

fits into the major groove of DNA (**Fig. 1**). Arg192 is located on the helix-2 and the side chain of Arg192 forms salt-bridges with Glu203 on the helix-3. The residues Arg192 and Glu203 are conserved in the homeodomain family and are considered to contribute to stability of the three helices in the homeodomain structure through the formation of salt bridge[16,20,21]. In addition, Arg192 of PAX4 is thought to bind with the phosphate backbone of double-stranded DNA (**Fig. 1**). The Arg residue corresponding to Arg192 of PAX4 is considered to be involved with DNA binding in other members of the homeodomain family[16,21]. Substitution of Arg192 by Ser or His disrupts the formation of the salt-bridges with Glu203 due to loss of the positively charged arginine side chain (**Fig. 2**), reducing the stability of the homeodomain. In addition, substitution of Arg192 by Ser or His is predicted to disrupt the direct binding with the phosphate backbone of double-stranded DNA (**Fig. 2**). In models of the two mutants (p.Arg192Ser and p.Arg192His) binding to DNA, the distance between the side chain of Ser or His and the phosphate backbone of DNA was 6.57Å and 6.15Å, respectively, indicating these residues could not interact with the phosphate backbone of DNA. Accordingly, the p.Arg192Ser and p.Arg192His variants of PAX4 are deduced to show decreased stability of the homeodomain containing the helices-2 and -3 involved with DNA binding, as well as loss of the ability to bind directly to the phosphate backbone of double-stranded DNA. Thus, these mutations are deduced to affect structural stability of the PAX4 homeodomain and its binding to DNA. It has been reported that missense mutations of the PAX3 homeodomain associated with Waardenburg syndrome are predicted to destabilize the homeodomain or affect DNA binding[16], in agreement with the structural influence of these two PAX4 variants.

Cell culture

INS-1 832/13 cells were maintained in RPMI 1640 medium supplemented with 10% heat-inactivated fetal bovine serum, 10 mM HEPES, 50 μM β-mercaptoethanol, 1 mM sodium pyruvate, 100 μg/ml streptomycin, and 100 IU/ml penicillin in humidified air containing 5% CO_2.

Quantitative PCR

Total RNA was obtained from INS-1 832/13 cells using the TaqMan Fast Advanced Cells-to-CT Kit (ThermoFisher SCIENTIFIC) and was reverse-transcribed into cDNA using the TaqMan Fast Advanced Cells-to-CT Kit (ThermoFisher SCIENTIFIC). cDNA was put into TaqMan Fast Advanced Master Mix (ThermoFisher SCIENTIFIC) containing TaqMan Gene

expression Assays (ThermoFisher SCIENTIFIC). The relative mRNA expression was calculated through normalization to the GAPDH mRNA level, according to the $2^{-\Delta\Delta Ct}$ method[22]. Experiments were repeated four times independently.

Statistical Analysis

The fold increase of the mRNA expression in the cells overexpressing PAX4 wild-type and variants compared with that in the cells transfected with empty vector was shown as mean ± s.e.m. The Student's t-test was used for statistical analyses, assuming equal variance, and p-values were calculated based on the 2-tailed test. Differences were statistically significant when the calculated p-value was less than 0.05.

References

15. Waterhouse, A. et al. SWISS-MODEL: homology modelling of protein structures and complexes. *Nucleic Acids Res.* **46**, W296-W303 (2018).

16. Birrane, G., Soni, A., & Ladias, J. A. Structural basis for DNA recognition by the human PAX3 homeodomain. *Biochemistry* **48**, 1148-1155 (2009).

17. Guex, N., & Peitsch, M. C. SWISS-MODEL and the Swiss-PdbViewer: an environment for comparative protein modeling. *Electrophoresis* **18**, 2714-2723 (1997).

18. Fuchsberger, C. et al. The genetic architecture of type 2 diabetes. *Nature* **536**, 41-47 (2016).

19. Kwak, S. H. et al. Nonsynonymous Variants in PAX4 and GLP1R Are Associated With Type 2 Diabetes in an East Asian Population. *Diabetes* **67**, 1892-1902 (2018).

20. Torrado, M. et al. Role of conserved salt bridges in homeodomain stability and DNA binding. *J. Biol. Chem.* **284**, 23765-23779 (2009).

21. Miyazono, K. et al. Cooperative DNA-binding and sequence-recognition mechanism of aristaless and clawless. *EMBO J.* **29**, 1613-1623 (2010).

22. Livak, K. J., & Schmittgen, T. D. Analysis of relative gene expression data using real-time quantitative PCR and the 2(-Delta Delta C(T)) Method. *Methods* **25**, 402-408 (2001).

Supplementary Table 1. Variants of genes involved in the MODY pathways associated with T2D in the Japanese population.

						Japanese				European §				MAF in 1000 Genomes Phase 3		
**LOCUS***	**rsID †**	**CHR**	**POS**	**RA**	**OA**	**MAF_{JPN}**	**P_{JPN}**	**OR_{JPN}**	**95%CI**	**MAF_{EUR}**	**P_{EUR}**	**OR_{EUR}**	**95%CI**	**EAS**	**JPT**	**EUR**
AGPAT9/NKX6-1	rs201597274	4	85,301,870	T	C	0.044	2.1E-12	1.17	1.12-1.23	ND	ND	ND	ND	0.020	0.034	0.0070
GCK	rs2908279	7	44,174,857	G	T	0.40	5.3E-12	1.07	1.05-1.09	0.50	1.1E-07	1.03	1.02-1.05	0.37	0.40	0.49
PAX4	rs2233580	7	127,253,550	T	C	0.092	4.1E-74	1.32	1.28-1.36	0.00050	2.9E-01	1.62	0.66-3.97	0.098	0.11	0
MNX1	rs1182389	7	157,038,803	G	A	0.46	1.1E-08	1.05	1.03-1.07	0.36	1.7E-11	1.05	1.03-1.06	0.47	0.47	0.37
HHEX/IDE	rs12219514	10	94,466,439	A	G	0.13	4.8E-39	1.18	1.15-1.21	0.44	4.6E-61	1.12	1.10-1.13	0.13	0.13	0.44
INS-IGF2_KCNQ1	rs2237897 ‡	11	2,858,546	C	T	0.38	2.6E-168	1.30	1.27-1.32	0.046	1.8E-31	1.21	1.17-1.25	0.35	0.39	0.047
HNF1A	rs187150787	12	121,327,809	A	G	0.037	6.0E-12	1.20	1.14-1.27	ND	ND	ND	ND	0.020	0.024	0
HNF1B	rs11651052	17	36,102,381	A	G	0.32	2.2E-34	1.12	1.10-1.14	0.47	8.9E-29	1.07	1.06-1.09	0.25	0.33	0.47
HNF4A	rs16988991	20	42,989,777	A	G	0.45	3.8E-09	1.05	1.04-1.07	0.16	5.8E-09	1.05	1.03-1.07	0.42	0.40	0.16

CHR, chromosome; POS, position in Human Genome version 19 (hg19), build 37; RA, risk allele; OR, other allele; MAF, minor allele frequency; OR, odds ratio; CI, confidence interval.

* Based on locus information reported by Suzuki et al. (*Nat. Genet.* 2019)[4]

† Variants at primary signals[4] without adjustment for BMI are shown. Information on the same variants from GWAS meta-analysis in a European population[3] without adjustment for BMI is also shown.

‡ The variant rs2237897 is located within 1 Mb of the lead independent variant rs4929965 at the *INS/IGF2* locus in Europeans[3], which is also associated with T2D in Japanese[4] (see Supplementary Table 2).

§ Based on summary statistics reported by Mahajan et al. (*Nat. Genet.* 2018)[3]

Supplementary Table 2. Variants of genes involved in the MODY pathway associated with T2D in Europeans.

						European				Japanese ‡				MAF in 1000 Genomes Phase 3		
**LOCUS***	**rsID †**	**CHR**	**POS**	**RA**	**OA**	**MAF_{EUR}**	**P_{EUR}**	**OR_{EUR}**	**95%CI**	**MAF_{JPN}**	**P_{JPN}**	**OR_{JPN}**	**95%CI**	**EAS**	**JPT**	**EUR**
SLC2A2	rs9873618	3	170,733,076	G	A	0.29	4.8E-21	1.07	1.05-1.08	0.20	1.0E-02	1.03	1.01-1.05	0.21	0.25	0.29
GCK	rs878521	7	44,255,643	A	G	0.25	1.9E-13	1.06	1.04-1.07	0.41	2.1E-02	1.02	1.00-1.04	0.35	0.36	0.25
MNX1	rs6459733	7	156,930,550	G	C	0.33	2.4E-17	1.06	1.05-1.07	0.46	2.7E-07	1.05	1.03-1.07	0.45	0.44	0.36
NEUROG3	rs2642588	10	71,466,578	G	T	0.30	2.2E-14	1.05	1.04-1.07	0.082	1.9E-01	1.02	0.99-1.06	0.13	0.077	0.30
HHEX/IDE	rs10882101	10	94,462,427	T	C	0.41	1.4E-08	1.06	1.04-1.08	0.29	1.2E-35	1.13	1.10-1.15	0.28	0.33	0.43
INS/IGF2	rs4929965	11	2,197,286	A	G	0.38	4.0E-26	1.07	1.06-1.09	0.081	3.1E-13	1.12	1.09-1.16	0.087	0.067	0.36
HNF1A	rs56348580	12	121,432,117	G	C	0.31	2.3E-13	1.05	1.04-1.07	ND	ND	ND	ND	0.0050	0	0.30
ONECUT1	rs2456530	15	53,091,553	T	C	0.13	5.4E-09	1.06	1.04-1.08	0.49	1.3E-02	1.02	1.00-1.04	0.50	0.46	0.11
HNF1B	rs10908278	17	36,099,952	T	A	0.48	6.4E-36	1.08	1.07-1.10	0.31	6.7E-32	1.13	1.10-1.15	0.23	0.32	0.48
NKX2.2	rs13041756	20	21,466,795	C	T	0.11	1.4E-08	1.06	1.04-1.08	0.20	8.8E-01	1.00	0.98-1.02	0.21	0.23	0.10
HNF4A	rs1800961	20	43,042,364	T	C	0.035	2.3E-22	1.18	1.15-1.23	0.010	7.7E-05	1.18	1.09-1.29	0.016	0.0048	0.037

CHR, chromosome; POS, position in Human Genome version 19 (hg19), build 37; RA, risk allele; OR, other allele; MAF, minor allele frequency; OR, odds ratio; CI, confidence interval.

* Based on locus information reported by Mahajan et al. (*Nat. Genet.* 2018)[3]

† Variants at primary signals[3] without adjustment for BMI are shown. Information on the same variants from GWAS meta-analysis in a Japanese population[4] without adjustment for BMI is also shown.

‡ Based on summary statistics reported by Suzuki et al. (*Nat. Genet.* 2019)[4]

Supplementary Table 3. Annotation of missense variants in *PAX4* with prediction tools.

Variant	Chr	Pos	Gene	Amino acid change	RA	OA	MAF in 1KGP			SIFT_pred	Polyphen2_HDIV_pred	PROVEAN_pred	MetaLR_pred	FATHMM_pred
							EAS	JPT	EUR					
rs2233580	7	127,253,550	*PAX4*	p.R192H	T	C	0.098	0.11	0.000	Deleterious	Probably damaging	Deleterious	Deleterious	Deleterious
rs3824004	7	127,253,551	*PAX4*	p.R192S	T	G	0.023	0.038	0.000	Deleterious	Probably damaging	Deleterious	Deleterious	Deleterious

CHR, chromosome; POS, position in Human Genome version 19 (hg19), build 37; RA, risk allele; OR, other allele; MAF, minor allele frequency.
